# Corrosion and microstructural analysis data for AISI 316L and AISI 347H stainless steels after exposure to a supercritical water environment

**DOI:** 10.1016/j.dib.2016.04.013

**Published:** 2016-04-11

**Authors:** A. Ruiz, T. Timke, A. van de Sande, T. Heftrich, R. Novotny, T. Austin

**Affiliations:** European Commission, Joint Research Centre (JRC), Institute for Energy and Transport (IET), Westerduinweg 3, 1755 LE Petten, Netherlands

**Keywords:** Supercritical water reactor, SCWR, General corrosion, Austenitic stainless steel, Microstructural analysis

## Abstract

This article presents corrosion data and microstructural analysis data of austenitic stainless steels AISI 316L and AISI 347H exposed to supercritical water (25 MPa, 550 °C) with 2000 ppb of dissolved oxygen. The corrosion tests lasted a total of 1200 h but were interrupted at 600 h to allow measurements to be made. The microstructural data have been collected in the grain interior and at grain boundaries of the bulk of the materials and at the superficial oxide layer developed during the corrosion exposure.

**Specifications Table**

**Table:** 

Subject area	*Physics, chemistry*
More specific subject area	*Materials science*
Type of data	*Image (scanning electron microscopy, SEM); chemical composition (Energy- dispersive X-ray spectroscopy, EDX); weight change*
How data was acquired	*Images taken in a Leo Supra50 SEM. Microanalysis acquired with an EDX detector from Oxford Instruments coupled to the SEM. Weight measurements were carried out in a Sartorius analytical balance.*
Data format	*Raw SEM images; EDX spectra analyzed using INCA software*
Experimental factors	*Samples exposed to a flow of 5* *dm^3^/h of supercritical water at 550 °C, 25* *MPa and 2000* *ppb dissolved oxygen during 600* *h and 1200* *h.*
Experimental features	*General corrosion tests were conducted according to ASTM standard G1-03 in a supercritical water (SCW) loop facility at the Institute for Energy and Transport of the Joint Research Centre (JRC IET). Tests were done in an autoclave with a flow of 5 dm^3^/h of SCW at 550 °C, 25 MPa and with 2000 ppb dissolved O*_2_[1]. *SEM-EDX analysis was performed on cross sections of the specimens and on the superficial top-view of the oxide layers. The microstructure was analyzed at the oxide layers and in the bulk of the materials (inside of the grains and at grain boundaries)*.
Data source location	*Petten, The Netherlands*
Data accessibility	*Data is within this article and available from*https://odin.jrc.ec.europa.eu

## Value of the data

• General corrosion tests in supercritical water environment are needed to progress in the design, analysis and licensing of a fuel assembly cooled with supercritical water in a research reactor [2].
• The austenitic stainless steels tested are candidate materials for structural components of the European Generation IV Supercritical Water reactor concept [3].
• Microstructural and chemical analysis of the oxide, bulk and grain boundaries may provide indications about materials performances and possible failure mechanisms.
• The microstructural characterization of the materials subjected to corrosion tests provides insight into the corrosion behavior of such materials and is of interest to industries and research bodies developing more performant materials to be used in harsh environments.

## Data

Original micrographs and chemical composition obtained from SEM–EDX are presented to show the materials microstructure and elementary composition at different places of the specimens after exposure to supercritical water.

Data of corrosion resistance is presented in a table summarizing the main features of weight change and oxide growth. These data are also open source in the ODIN database (https://odin.jrc.ec.europa.eu).

## Experimental design, materials and methods

AISI 316L and AISI 347H specimens were subjected to general corrosion tests in SCW using the parameters described above. The corrosion coupons were cleaned with ethanol in an ultrasonic bath, dried, weighted and then attached to a specimen holder rack using 316L wires. Several coupons of each material were placed into the autoclave, and then one coupon at a time was removed after 600 h and after 1200 h for microstructural analysis. The nominal chemical composition before exposure, as well as the weight change and the oxide thickness of 316L and 347H at the different exposure times can be found in [4], [5].

For SEM–EDX analysis, the specimens were cut and mounted in Bakelite. They were then polished with #1200 emery paper and etched in SuperVillella׳s solution. SEM imaging was done at 15 kV EHT and using secondary electrons. EDX compositional analysis was performed at different places of the oxide layers, the bulk and the grain boundaries of the materials using an acceleration voltage of 15 kV, 60 µm aperture and lifetime of 60 s. The EDX spectra were analyzed with the INCA software and the data of the chemical composition are presented normalized to 100%.

The 316L grain size was between 10 µm and 50 µm, as measured in SEM images. 347H presented slightly larger grains, of sizes between 20 µm and 100 µm. For both materials, the grain size did not change significantly during the exposures.

## Microstructural analysis of AISI 316L

**Fig. 1:**
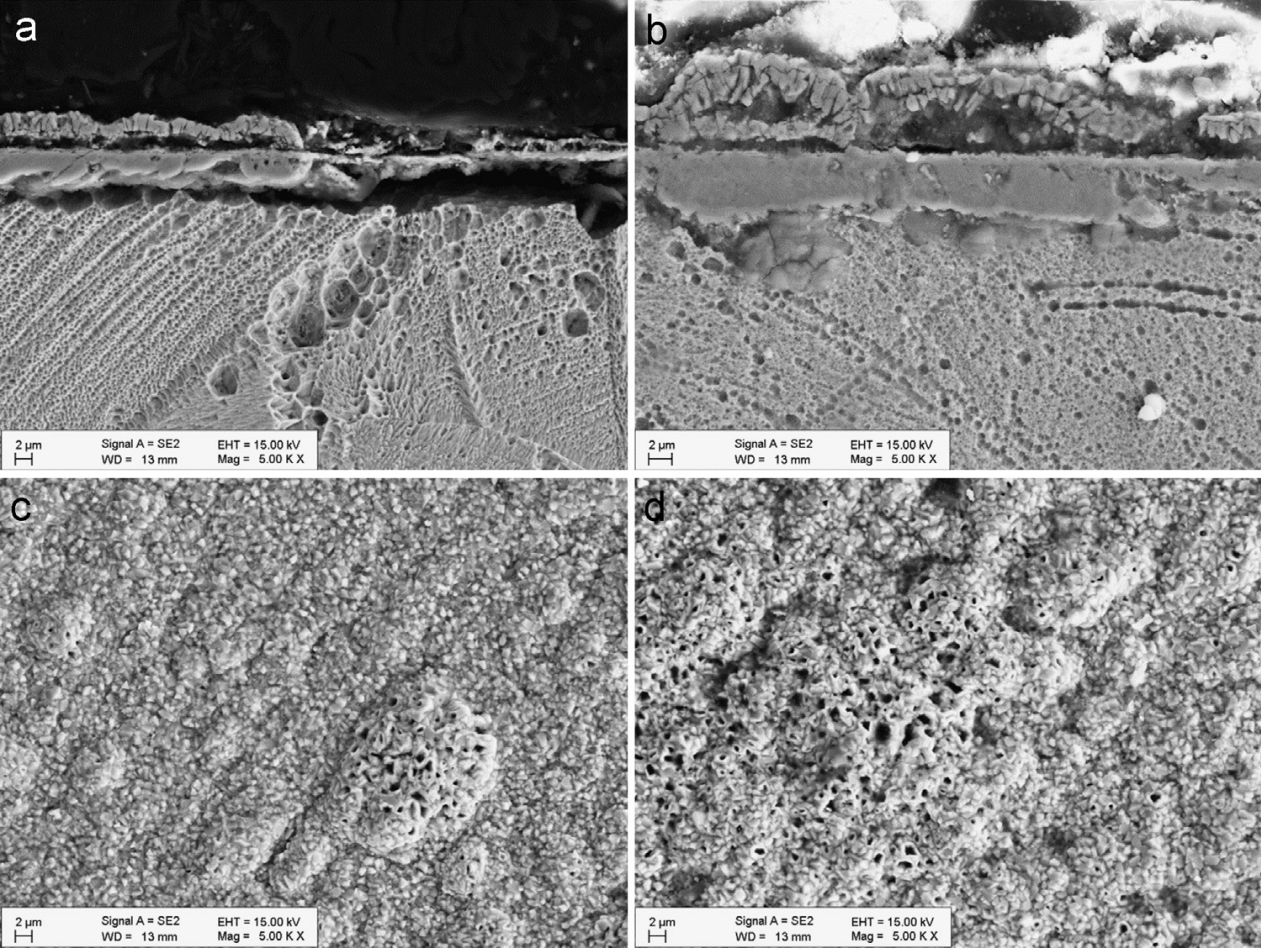
Cross-section and surface-view SEM images of the oxide layer formed on 316L after 600 h (a, c) and 1200 h (b, d) exposure to SCW.

Fig. 1 shows the structure of the oxide layer after 600 h and 1200 h of exposure. The cross section images (a and b) reveal the formation of a dual layer. The morphology of the upper oxide layer is seen in the surface-view images (c and d). At 600 h the oxide layer was mostly flat, while increased oxide porosity was observed only in the thicker regions. At 1200 h the oxide layer was rougher and there were more porous regions.

**Fig. 2:**
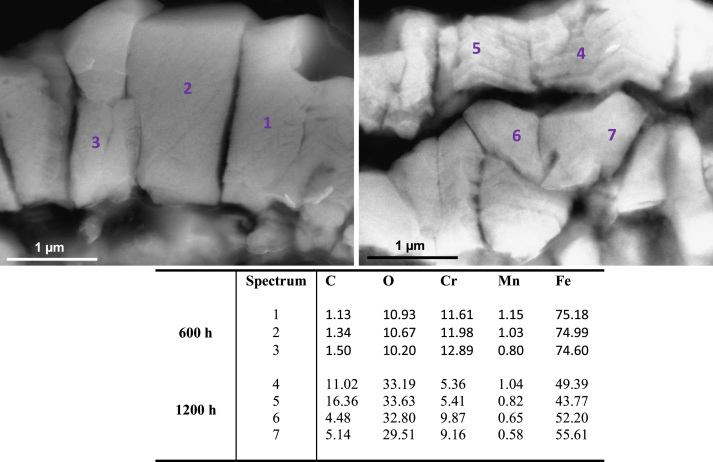
SEM images and composition table (in wt%) at several points of the upper layer of the oxide bilayer formed on 316L after 600 h (left image) and 1200 h (right image) exposure to SCW.

**Fig. 3:**
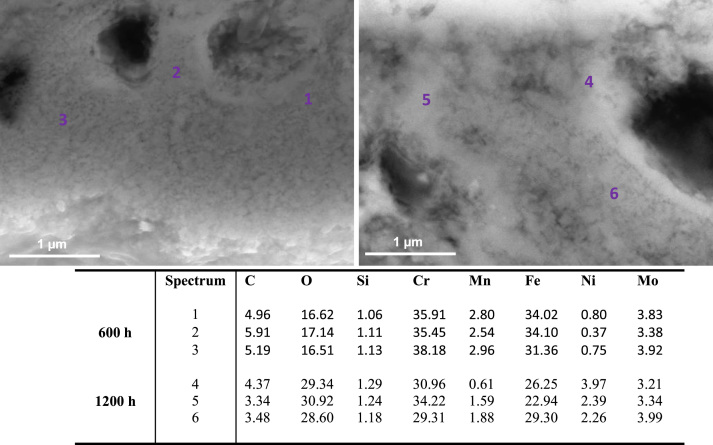
SEM images and composition table (in wt%) at 3 points of the lower layer of the oxide bilayer formed on 316L after 600 h (left image) and 1200 h (right image) exposure to SCW.

**Fig. 4:**
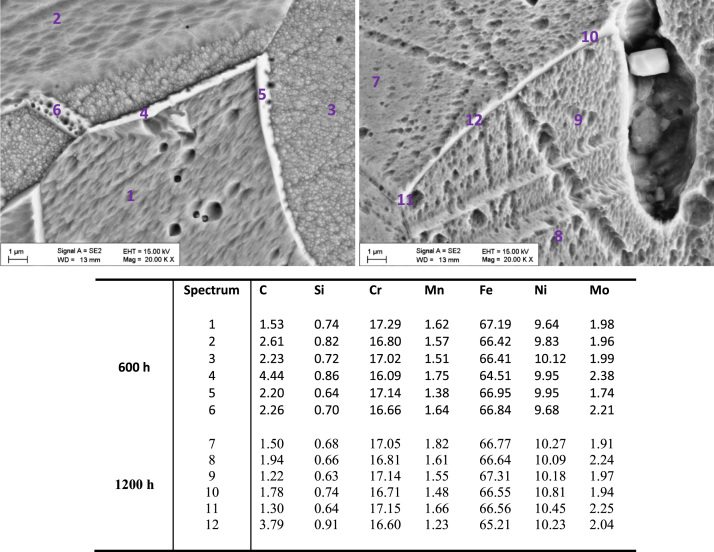
SEM images and composition table (in wt%) of the bulk material (spectra 1–3, 7–9) and the grain boundaries (spectra 4–6, 10–12) of 316L after 600 h (spectra 1–6) and after 1200 h (spectra 7–12) exposure to SCW.

Fig. 2, Fig. 3 show SEM images and elementary composition of the upper (Fig. 2) and lower (Fig. 3) layers of the dual oxide layer developed on the surface of 316L coupons after 600 h and 1200 h exposure. As evidenced by the tabulated data, the concentration of oxygen increases with the exposure time. Fig. 4 shows images of the bulk of 316L. Segregated particles were not found in the bulk or at the grain boundaries. EDX spectra were acquired at different points of the bulk and on the grain boundaries of the material after exposure. The elementary composition in the bulk and at grain boundaries did not significantly change during corrosion.

## Microstructural analysis of AISI 347H

**Fig. 5:**
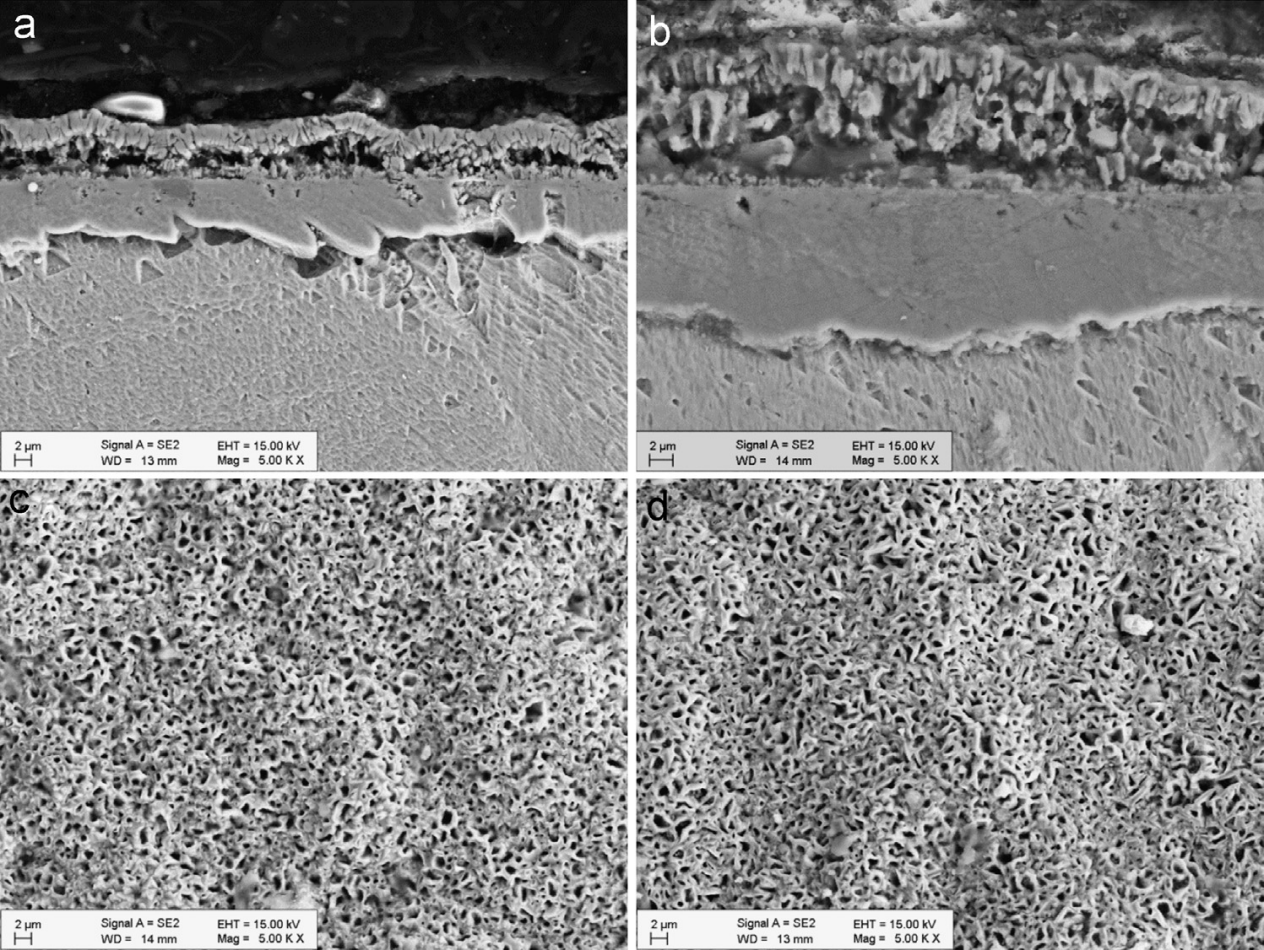
Cross-section and surface-view SEM images of the oxide layer formed on 347H after 600 h (a, c) and 1200 h (b, d) exposure to SCW.

Fig. 5 shows the structure of the oxide layer after 600 h and 1200 h of exposure. Similar to 316L, the cross-section images of 347H specimens (a and b) also reveal the formation of a dual layer. The morphology of the upper oxide layer (c and d) is more homogeneous than in the case of 316L and the porosity is more evenly distributed.

**Fig. 6:**
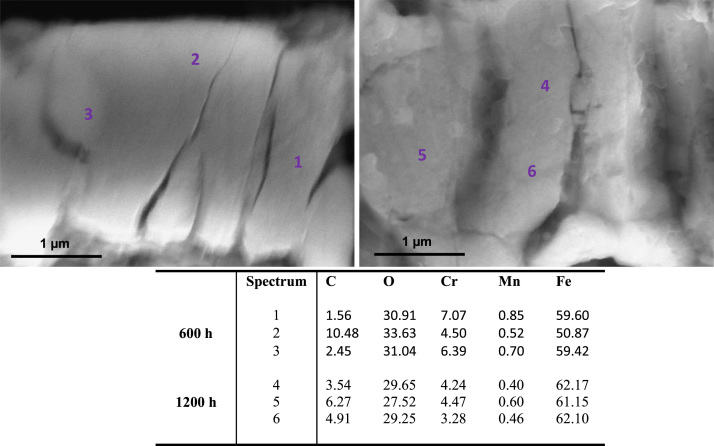
SEM images and composition table (in wt%) at several points of the upper layer of the oxide bilayer formed on 347H after 600 h (left image) and 1200 h (right image) exposure to SCW.

**Fig. 7:**
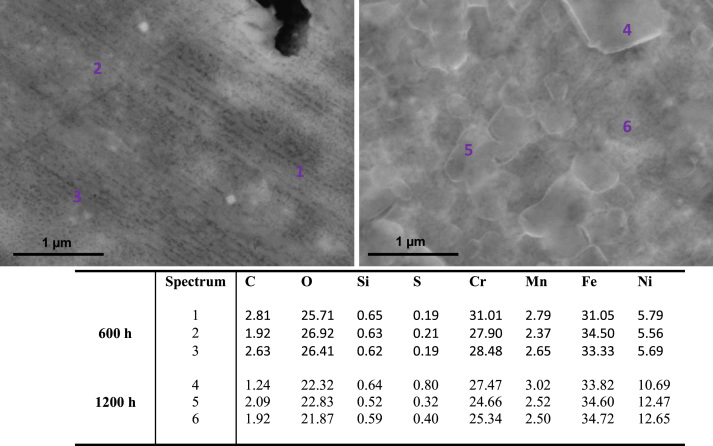
SEM images and composition table (in wt%) of the lower layer of the oxide bilayer formed on 347H after 600 h (left image) and 1200 h (right image) exposure to SCW.

**Fig. 8:**
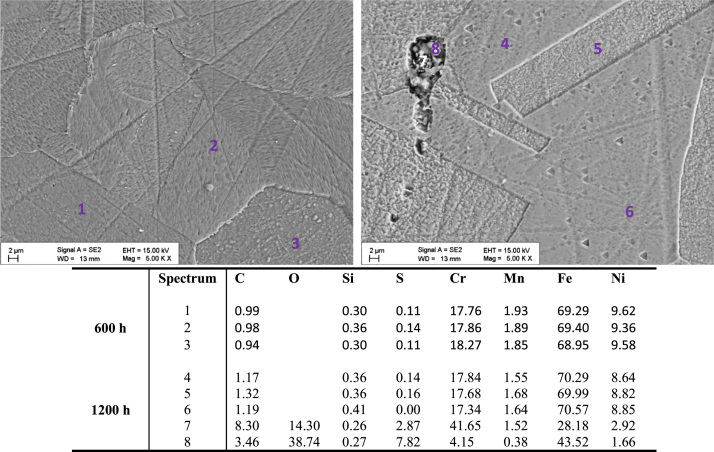
SEM images and composition table (in wt%) of the 347H bulk after 600 h (spectra 1–3) and after 1200 h (spectra 4–8) exposure to SCW. Oxide particles (spectra 4, 5) are found after 1200 h.

**Fig. 9:**
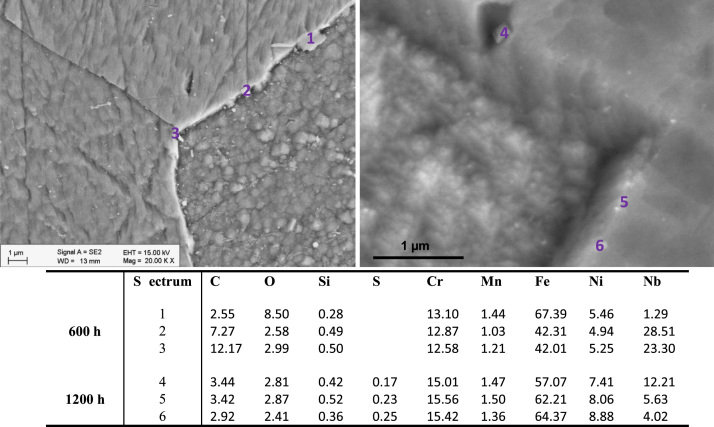
SEM images and composition table (in wt%) of the 347H grain boundaries after 600 h (spectra 1–3) and after 1200 h (spectra 4–6) exposure to SCW.

Fig. 6, Fig. 7 show SEM images and elementary composition of the upper (Fig. 6) and lower (Fig. 7) layers of the oxide dual layer developed on the surface of 347H coupons after 600 h and 1200 h exposure. As evidenced by the tabulated data, the concentration of oxygen slightly decreased with the exposure time. Fig. 8 shows images of the bulk of 347H. Some segregated particles are distinguished in the bulk and at grain boundaries. EDX spectra were acquired at different points of the bulk and also on two particles present in the material exposed 1200 h. The particles segregated in the bulk were rich in oxygen and sulfur. The composition of grain boundaries is shown in Fig. 9. Niobium-rich grain boundaries were detected at both exposure times.
