# Supplementary material for: Corrosion and microstructural analysis data for AISI 316L and AISI 347H stainless steels after exposure to a supercritical water environment
Source: Data Brief. 2016 Apr 11;7:1341–8. doi: 10.1016/j.dib.2016.04.013 (PMC4845079; doi:10.1016/j.dib.2016.04.013)
Supplement: Supplementary file 1 — Supplementary material [file mmc1.pdf]

Data article

Title: ***Corrosion and microstructural analysis data for AISI 316L and AISI 347H stainless steels after exposure to a supercritical water environment***

Authors: A. Ruiz, T. Timke, A. van de Sande, T. Heftrich, R. Novotny, T. Austin

Affiliations: European Commission, Joint Research Centre (JRC), Institute for Energy and Transport (IET). Westerduinweg 3, 1755 LE, Petten, Netherlands

Conflicts of interest: none

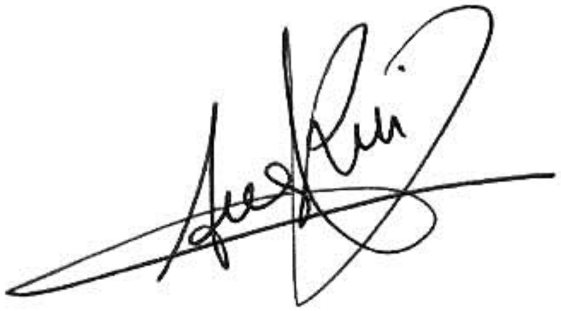

Corresponding author: AM. Ruiz Moreno

Contact email: ana.ruiz-moreno@ec.europa.eu
